# Supplementary material for: Coupling Machine Learning and High Throughput Multiplex Digital PCR Enables Accurate Detection of Carbapenem-Resistant Genes in Clinical Isolates
Source: Front Mol Biosci. 2021 Nov 23;8:775299. doi: 10.3389/fmolb.2021.775299 (PMC8650054; doi:10.3389/fmolb.2021.775299)
Supplement: Supplementary file 1 [file DataSheet1.pdf]

Supplementary Material

1 Supplementary Data

1.1 Supplementary Data S1

Experimental condition for Real-Time PCR: Each amplification reaction was performed in 10 μL of final volume with 5 μL of 2× SsoFast EvaGreen Supermix with Low ROX (BioRad, UK), 3 μL of PCR-grade water, 1 μL of 10× multiplex PCR primer mixture containing the five primer sets (5μM of each primer), and 1μL of different concentrations of synthetic DNA, clinical sample or controls. The reaction consisted of 10 min at 95 °C, followed by 45 cycles at 95 °C for 20 s, 67 °C for 45 s, and 72 °C for 30 s. Melting curve analysis was performed with one cycle at 65 °C for 60 s and reading from 65 to 97 °C with an increment of 0.2 °C. The PCR machine used in this study was the Light Cycler 96 real-time PCR system (Roche Diagnostics, Germany).

1.2 Supplementary Data S2

AMCA coefficient equation: As the colourmap in Supplementary Figure S10 shows, the Amplification Curve Analysis (ACA) and Melting Curve Analysis (MCA) coefficients contribute differently to the target classification of the Amplification and Melting Curve Analysis (AMCA) model. More specifically, because the AMCA is a supervised linear model, the coefficients can be investigated to understand how it weighs the predictions from ACA and MCA. The output of AMCA is defined by:

$$y = \widehat{W}_{ACA}\, y_{ACA} + \widehat{W}_{MCA}\, y_{MCA}$$

where $y_{ACA} \in \mathbb{R}^5$ and $y_{MCA} \in \mathbb{R}^5$ are the probabilities outputted from the ACA and MCA models, respectively, and $\widehat{W}_{ACA} \in \mathbb{R}^{5X5}$ and $\widehat{W}_{MCA} \in \mathbb{R}^{5X5}$ are the model coefficients relating to ACA and MCA, respectively.

2 Supplementary Figures and Tables

2.1 Supplementary Figures

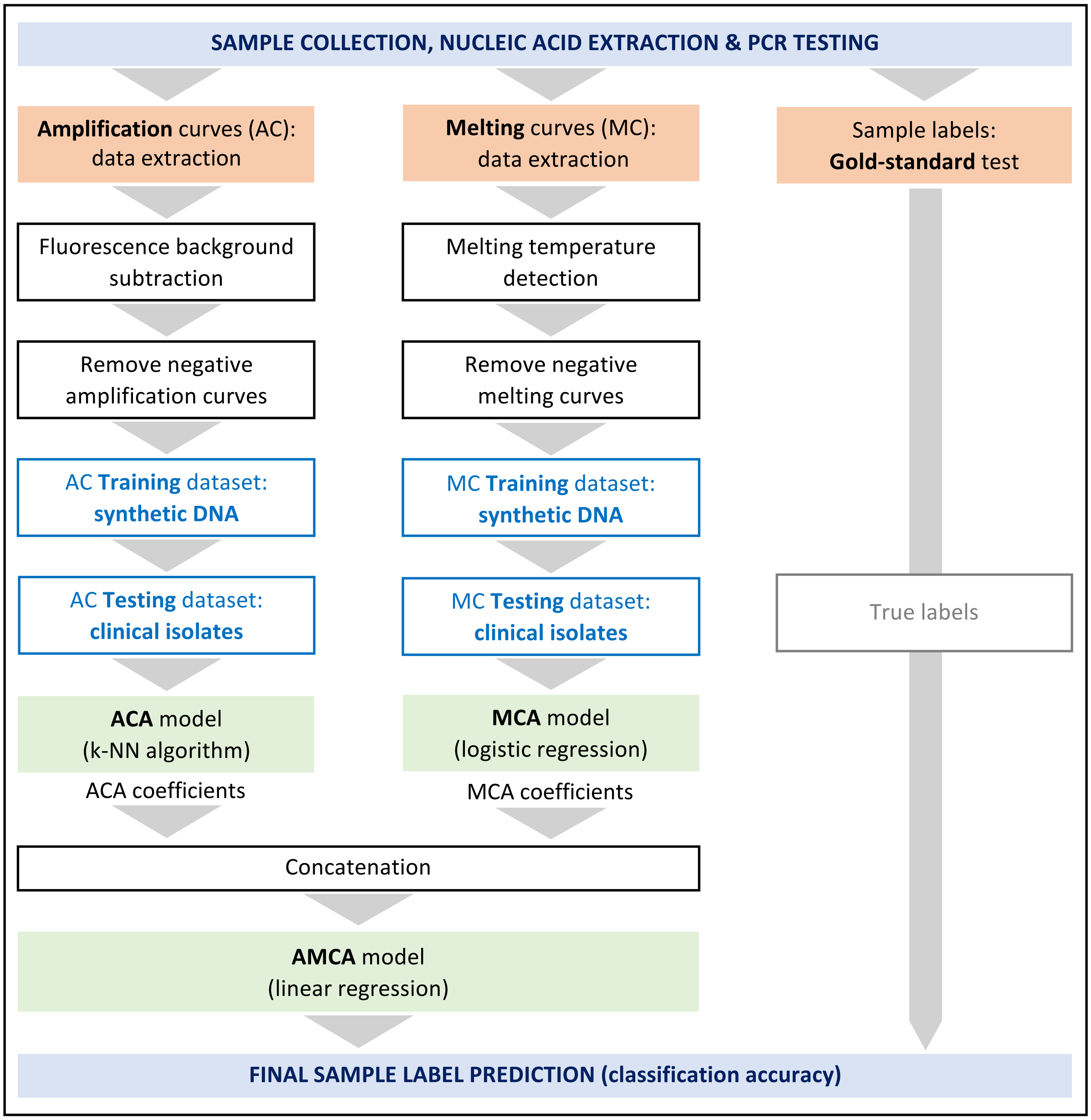

Supplementary Figure S1. Flowchart to visualize the data processing workflow for the AMCA method. The true label (determined by a gold standard PCR method such as Cepheid Xpert Carba-R assay) are only required as comparison for the predicated labels from the AMCA algorithm. The training data set used for the ACA and MCA model is generated using synthetic DNA with known labels and concentrations, whereas the testing dataset is generated from clinical isolates dataset using digital real-time PCR. The output of ACA and MCA models are probabilities for each target and their coefficients are concatenated as input of the final AMCA model.

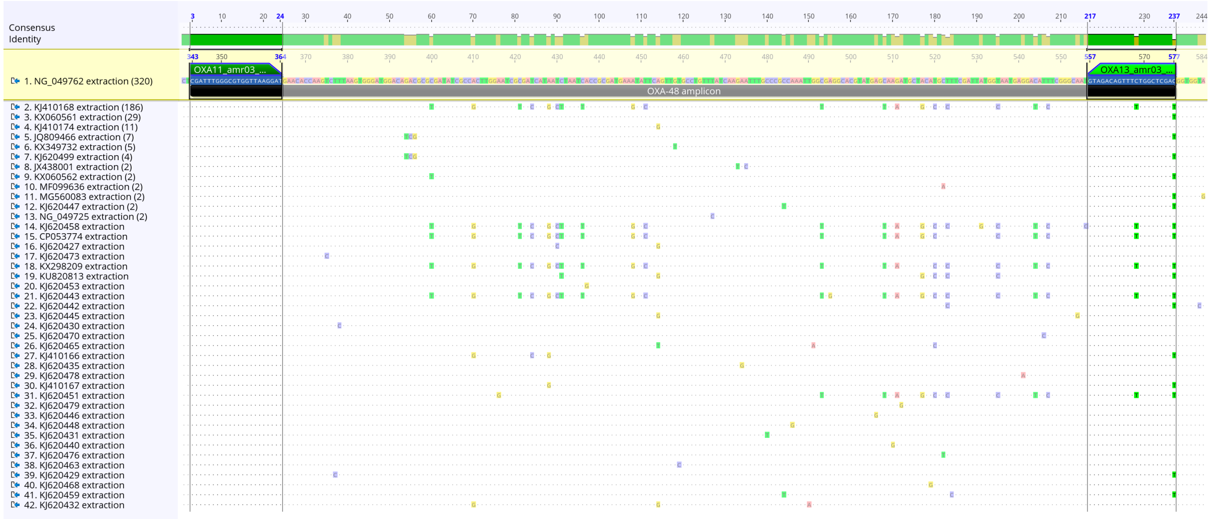

Supplementary Figure S2. *Inclusivity alignment of bla*$_{\text{OXA-48}}$. Sequences retrieved from nr/nt NCBI database (n = 603) with a coverage of 100% for each primer binding region. The alignment shows only unique sequences that differ from the reference NG_049762 in the amplification region. The sequences are ordered from the largest number of unique sequences to the lowest as shown in bracket (after the NCBI accession number) on the left side of the alignment.

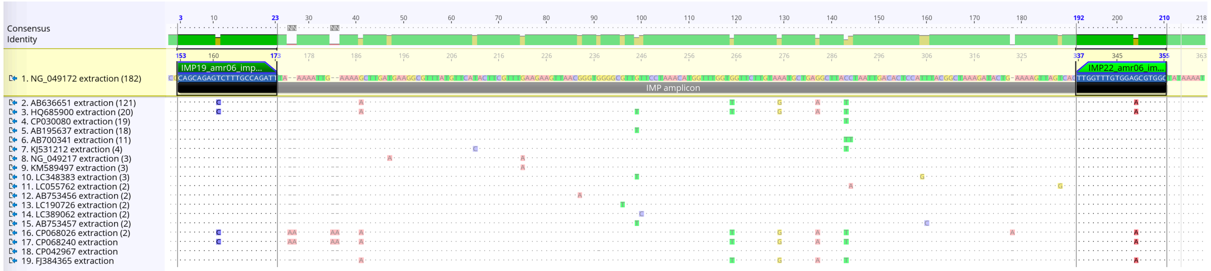

Supplementary Figure S3. *Inclusivity alignment of bla*$_{\text{IMP}}$ (including bla$_{\text{IMP-1}}$ and bla$_{\text{IMP-4}}$ groups). Sequences retrieved from nr/nt NCBI database (n = 400) with a coverage of 100% for each primer binding region. The alignment shows only unique sequences that differ from the reference NG_049172 in the amplification region. The sequences are ordered from the largest number of unique sequences to the lowest as shown in bracket (after the NCBI accession number) on the left side of the alignment.

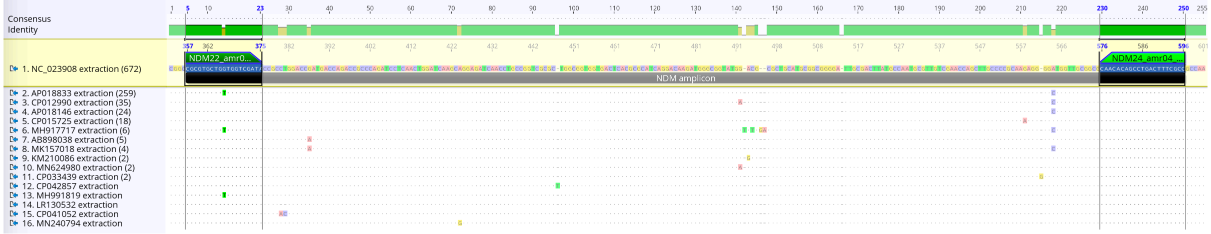

Supplementary Figure S4. *Inclusivity alignment of bla*$_{\text{NDM}}$. Sequences retrieved from nr/nt NCBI database (n = 1035) with a coverage of 100% for each primer binding region. The alignment shows only unique sequences that differ from the reference NC_023908 in the amplification region. The sequences are ordered from the largest number of unique sequences to the lowest as shown in bracket (after the NCBI accession number) on the left side of the alignment.

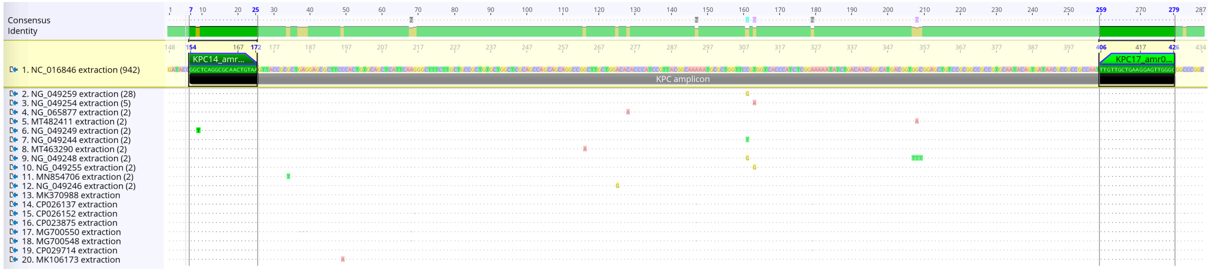

Supplementary Figure S5. *Inclusivity alignment of bla*$_{\mathrm{KPC}}$. Sequences retrieved from nr/nt NCBI database (n = 1001) with a coverage of 99.9% for each primer binding region. The alignment shows only unique sequences that differ from the reference NC_016846 in the amplification region. The sequences are ordered from the largest number of unique sequences to the lowest as shown in bracket (after the NCBI accession number) on the left side of the alignment.

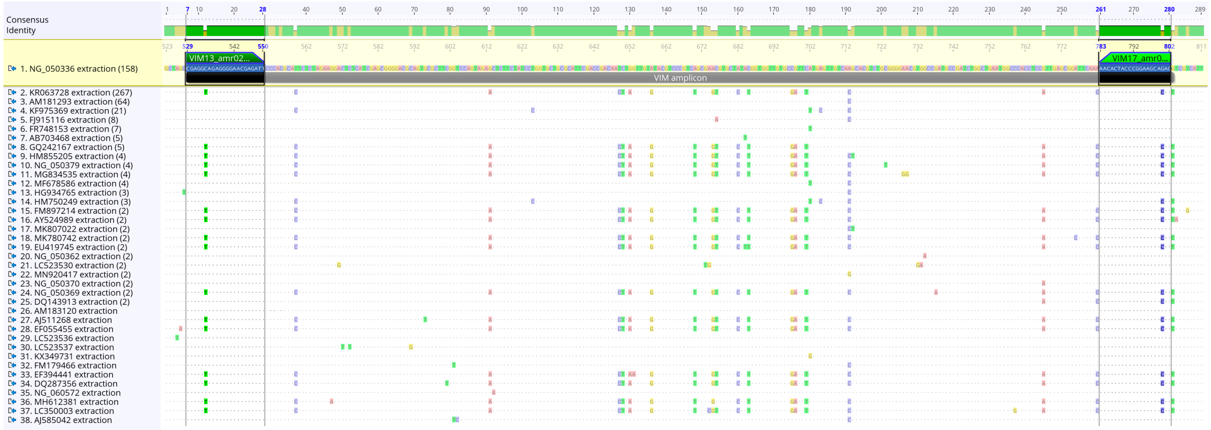

Supplementary Figure S6. *Inclusivity alignment of bla*$_{\mathrm{VIM}}$. Sequences retrieved from nr/nt NCBI database (n = 593) with a coverage of 100% for each primer binding region. The alignment shows only unique sequences that differ from the reference NG_050336 in the amplification region. The sequences are ordered from the largest number of unique sequences to the lowest as shown in bracket (after the NCBI accession number) on the left side of the alignment.

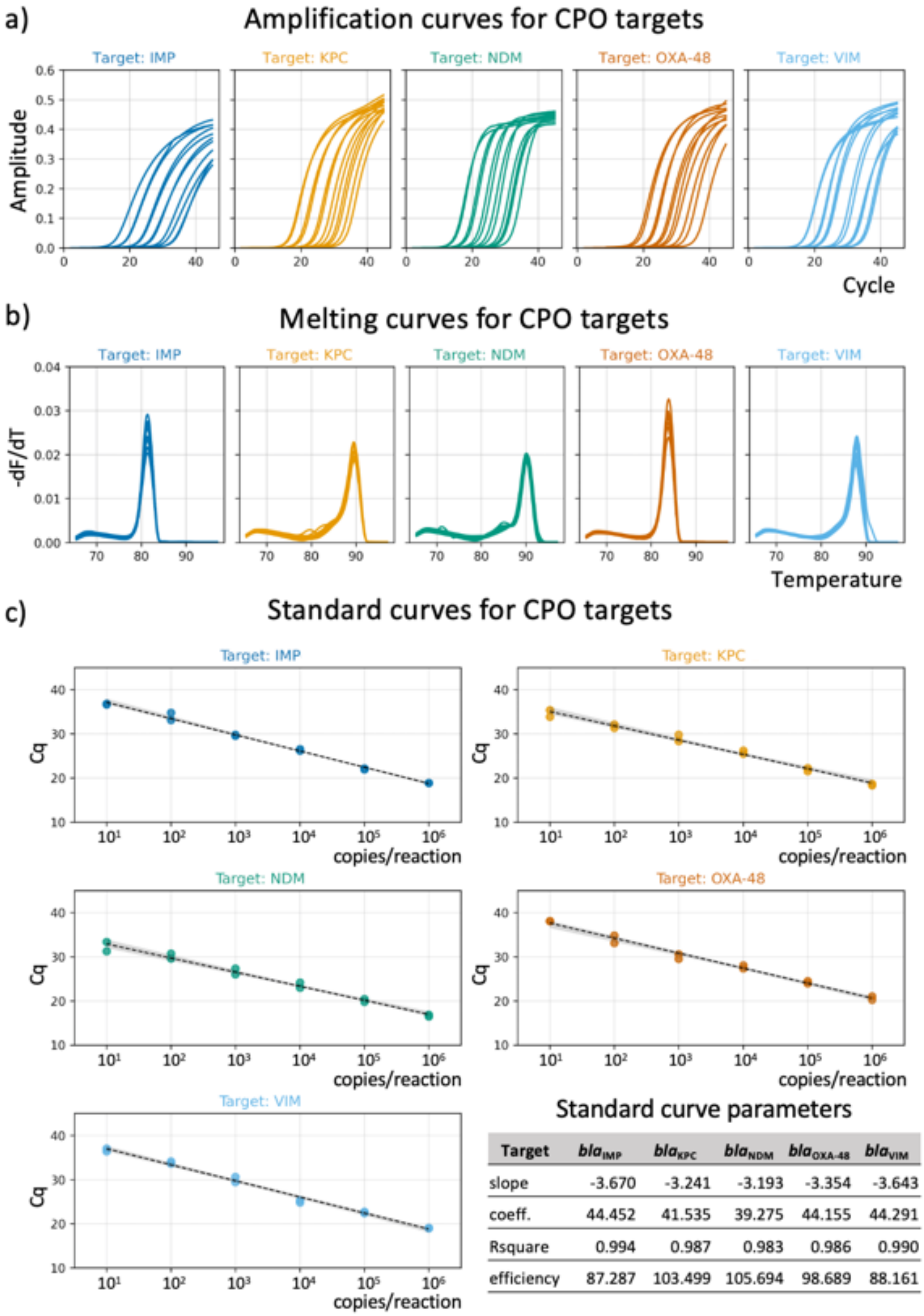

Target	*bla*$_{IMP}$	*bla*$_{KPC}$	*bla*$_{NDM}$	*bla*$_{OXA-48}$	*bla*$_{VIM}$
slope	-3.670	-3.241	-3.193	-3.354	-3.643
coeff.	44.452	41.535	39.275	44.155	44.291
Rsquare	0.994	0.987	0.983	0.986	0.990
efficiency	87.287	103.499	105.694	98.689	88.161

Supplementary Figure S7. Analysis of real-time amplification and melting curves from qPCR instruments. (a) Raw real-time amplification curves. (b) Raw melting curve for each target. (c) Standard curves for each target using our new 5-plex PCR assay. (Bottom Panel) Table with relevant meta data for each standard curve.

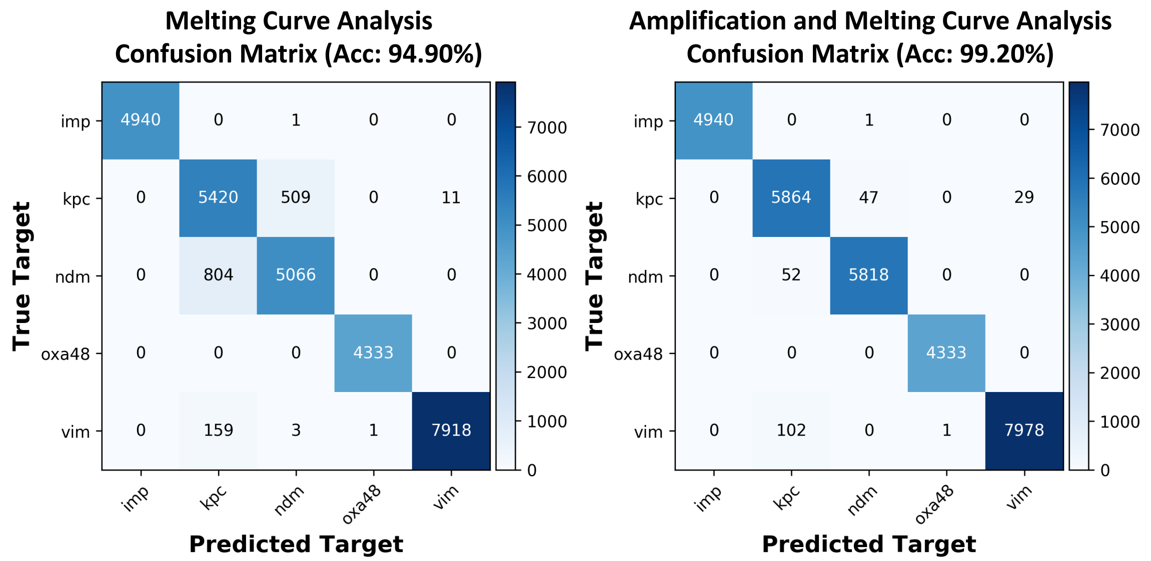

Supplementary Figure S8. *Performance of the MCA and AMCA methods for multiplexing the five carbapenemase gene targets in the training dataset using synthetic DNA templates*. (left) Confusion matrix illustrating the predictions from Melting Curve Analysis (MCA) algorithm. (right) Confusion matrix illustrating the predictions from Amplification and Melting Curve Analysis (AMCA) algorithm. Values in the matrices indicate the number of positive amplification events (N=29,165) with diagonal entries corresponding to correct predictions.

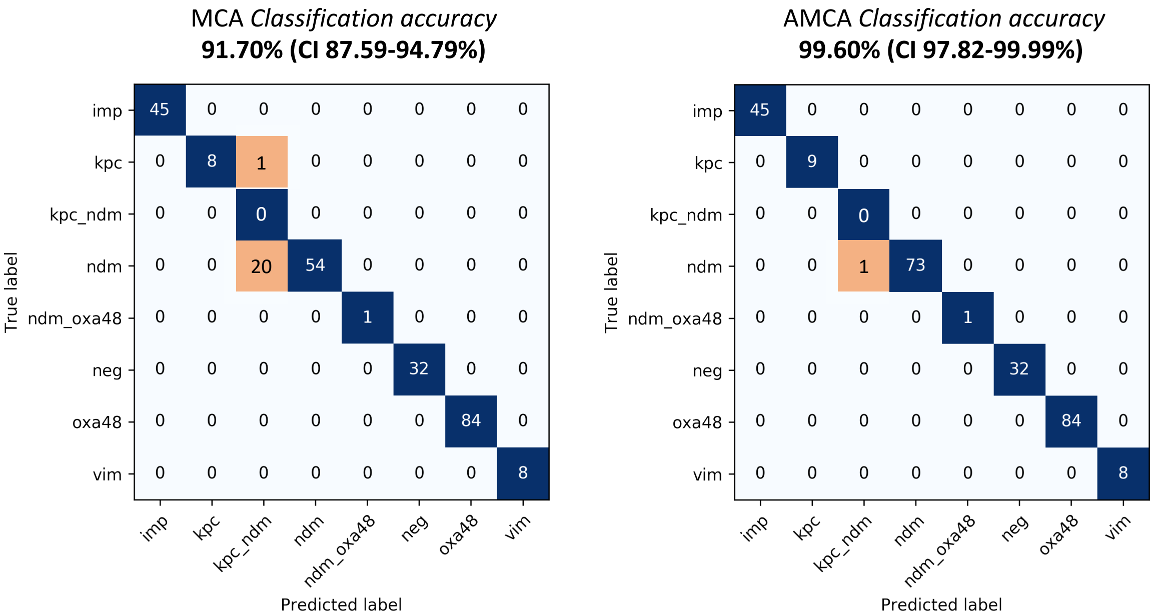

Supplementary Figure S9. *Performance of MCA and AMCA methods for multiplexing the five carbapenemase gene targets in clinical isolates*. (left) Confusion matrix illustrating the predictions from Melting Curve Analysis (MCA) algorithm. (right) Confusion matrix illustrating the predictions from Amplification and Melting Curve Analysis (AMCA) algorithm. Values indicate the number of clinical isolates (N=253) with diagonal entries corresponding to correct predictions.

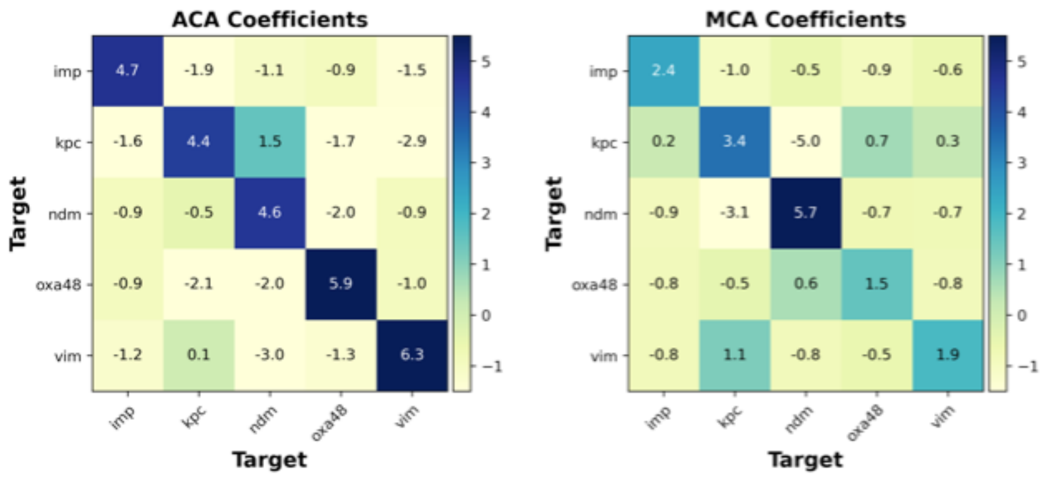

Supplementary Figure S10. *The coefficients of the AMCA model*. The values in the confusion matrices, ranging from -5 to 6.3, indicate the predictions weights from the ACA and MCA methods, respectively. Darker colours indicate more positive weighting. For example, as it can be observed, the AMCA weighs the prediction from ACA more heavily for *bla*$_{\mathrm{IMP}}$ target (4.7 in the ACA model compared to the 2.4 of the MCA model), instead for *bla*$_{\mathrm{NDM}}$ the situation is the opposite (5.7 in the MCA model compared to the 4.6 of the ACA model).

2.2 Supplementary Tables

Supplementary Tables S1. Bacterial isolates used in this study

Sample number	Species (MALDI-TOF)	Sample source	Collection date	Carbapene-mase gene carried	AMCA classification	Conc. (cp/µl)
CPO001	*Acinetobacter baumannii*	Bronchoalveolar lavage	26/02/2015	oxa23	neg	0.00E+00
CPO002	*Klebsiella pneumoniae*	Urine	15/04/2013	neg	neg	0.00E+00
CPO003	*Klebsiella pneumoniae*	Rectal swab	29/05/2018	neg	neg	0.00E+00
CPO004	*Enterobacter cloacae*	Right leg tissue	13/07/2018	neg	neg	0.00E+00
CPO005	*Escherichia coli*	Sputum	10/10/2015	neg	neg	0.00E+00
CPO006	*Escherichia coli*	Urine	04/01/2016	neg	neg	0.00E+00
CPO007	*Klebsiella pneumoniae*	Urine	29/02/2016	neg	neg	0.00E+00
CPO008	*Citrobacter freundii*	Urine	09/03/2016	neg	neg	0.00E+00
CPO009	*Klebsiella pneumoniae*	Urine	10/07/2016	neg	neg	0.00E+00
CPO010	*Enterobacter cloacae*	Bronchoalveolar lavage	15/08/2016	neg	neg	0.00E+00
CPO011	*Klebsiella pneumoniae*	Perineum	05/10/2016	neg	neg	0.00E+00
CPO012	*Klebsiella pneumoniae*	Right leg tissue	23/10/2016	neg	neg	0.00E+00
CPO013	*Klebsiella pneumoniae*	Rectal swab	26/12/2016	neg	neg	0.00E+00
CPO014	*Escherichia coli*	Urine	18/06/2017	neg	neg	0.00E+00
CPO015	*Klebsiella pneumoniae*	Urine	18/06/2017	neg	neg	0.00E+00
CPO016	*Klebsiella pneumoniae*	Urine	05/08/2017	neg	neg	0.00E+00
CPO017	*Enterobacter cloacae*	Sputum	18/08/2017	neg	neg	0.00E+00
CPO018	*Escherichia coli*	Urine	18/08/2017	neg	neg	0.00E+00
CPO019	*Klebsiella pneumoniae*	Urine	27/10/2017	neg	neg	0.00E+00

CPO020	*Klebsiella pneumoniae*	Urine	05/01/2018	neg	neg	0.00E+00
CPO021	*Enterobacter cloacae*	Wound swab	14/11/2017	neg	neg	0.00E+00
CPO022	*Escherichia coli*	Rectal swab	22/01/2018	neg	neg	0.00E+00
CPO023	*Escherichia coli*	Urine	22/01/2018	neg	neg	0.00E+00
CPO024	*Klebsiella pneumoniae*	Urine	26/01/2018	neg	neg	0.00E+00
CPO025	*Enterobacter spp*	Rectal swab	16/05/2019	neg	neg	0.00E+00
CPO026	*Escherichia coli*	Rectal swab	16/05/2019	neg	neg	0.00E+00
CPO027	*Enterobacter cloacae*	Rectal swab	12/05/2018	neg	neg	0.00E+00
CPO028	*Escherichia coli*	Rectal swab	22/04/2018	neg	neg	0.00E+00
CPO029	*Klebsiella pneumoniae*	Rectal swab	07/01/2019	neg	neg	0.00E+00
CPO030	*Pseudomonas aeruginosa*	Rectal swab	12/01/2019	neg	neg	0.00E+00
CPO031	*Escherichia coli*	Rectal swab	11/02/2019	neg	neg	0.00E+00
CPO032	*Acinetobacter baumannii*	Rectal swab	13/03/2019	neg	neg	0.00E+00
CPO033	*Pseudomonas aeruginosa*	Throat swab	31/03/2015	imp	imp	2.42E+03
CPO034	*Escherichia coli*	Rectal swab	12/05/2018	imp	imp	1.64E+04
CPO035	*Klebsiella pneumoniae*	Rectal swab	18/05/2018	imp	imp	2.06E+03
CPO036	*Enterobacter cloacae*	Rectal swab	23/06/2018	imp	imp	9.88E+01
CPO037	*Klebsiella pneumoniae*	Rectal swab	03/07/2018	imp	imp	8.00E+02
CPO038	*Escherichia coli*	Rectal swab	13/01/2019	imp	imp	3.24E+04
CPO039	*Klebsiella pneumoniae*	Rectal swab	14/01/2019	imp	imp	3.28E+04
CPO040	*Escherichia coli*	Rectal swab	30/01/2019	imp	imp	1.36E+04
CPO041	*Enterobacter cloacae*	Rectal swab	27/07/2019	imp	imp	2.11E+02

CPO042	*Klebsiella pneumoniae*	Rectal swab	24/07/2019	imp	imp	9.11E+03
CPO043	*Klebsiella pneumoniae*	Rectal swab	29/07/2019	imp	imp	3.69E+03
CPO044	*Enterobacter cloacae*	Rectal swab	26/08/2019	imp	imp	6.58E+03
CPO045	*Enterobacter cloacae*	Rectal swab	18/08/2019	imp	imp	1.67E+04
CPO046	*Klebsiella pneumoniae*	Rectal swab	06/05/2019	imp	imp	2.59E+04
CPO047	*Enterobacter cloacae*	Rectal swab	09/05/2019	imp	imp	1.46E+02
CPO048	*Enterobacter cloacae*	Rectal swab	11/05/2019	imp	imp	1.75E+04
CPO049	*Enterobacter spp*	Rectal swab	13/06/2019	imp	imp	3.27E+04
CPO050	*Escherichia coli*	Rectal swab	20/06/2019	imp	imp	9.15E+03
CPO051	*Enterobacter cloacae*	Rectal swab	09/09/2017	imp	imp	2.32E+04
CPO052	*Klebsiella pneumoniae*	Rectal swab	03/10/2017	imp	imp	4.27E+01
CPO053	*Enterobacter cloacae*	Rectal swab	05/10/2017	imp	imp	2.18E+04
CPO054	*Enterobacter spp*	Rectal swab	11/10/2017	imp	imp	1.60E+04
CPO055	*Citrobacter freundii*	Rectal swab	28/10/2017	imp	imp	5.55E+02
CPO056	*Klebsiella pneumoniae*	Rectal swab	22/01/2018	imp	imp	1.20E+03
CPO057	*Klebsiella pneumoniae*	Rectal swab	28/01/2018	imp	imp	9.89E+02
CPO058	*Enterobacter cloacae*	Urine	06/02/2018	imp	imp	7.53E+01
CPO059	*Enterobacter spp*	Rectal swab	21/02/2018	imp	imp	7.92E+02
CPO060	*Klebsiella pneumoniae*	Rectal swab	10/09/2018	imp	imp	1.89E+02
CPO061	*Enterobacter cloacae*	Rectal swab	07/09/2018	imp	imp	1.83E+02
CPO062	*Escherichia hermannii*	Rectal swab	20/11/2018	imp	imp	1.47E+02
CPO063	*Escherichia coli*	Rectal swab	21/02/2018	imp	imp	1.05E+03

CPO064	*Enterobacter cloacae*	Rectal swab	28/01/2018	imp	imp	1.20E+02
CPO065	*Klebsiella pneumoniae*	Rectal swab	02/04/2018	imp	imp	3.48E+04
CPO066	*Klebsiella pneumoniae*	Rectal swab	22/04/2018	imp	imp	3.45E+04
CPO067	*Enterobacter cloacae*	Rectal swab	18/08/2018	imp	imp	1.05E+02
CPO068	*Klebsiella pneumoniae*	Rectal swab	07/01/2019	imp	imp	1.72E+02
CPO069	*Pseudomonas aeruginosa*	Rectal swab	12/01/2019	imp	imp	4.95E+03
CPO070	*Enterobacter cloacae*	Rectal swab	11/02/2019	imp	imp	2.41E+02
CPO071	*Klebsiella pneumoniae*	Rectal swab	13/03/2019	imp	imp	4.20E+02
CPO072	*Escherichia hermannii*	Rectal swab	27/05/2019	imp	imp	3.01E+02
CPO073	*Enterobacter spp*	Rectal swab	08/05/2019	imp	imp	1.32E+02
CPO074	*Klebsiella pneumoniae*	Swab	11/05/2019	imp	imp	3.45E+01
CPO075	*Enterobacter cloacae*	Rectal swab	16/06/2016	imp	imp	5.43E+01
CPO076	*Enterobacter cloacae*	Rectal swab	16/05/2019	imp	imp	2.41E+04
CPO077	*Enterobacter cloacae*	Rectal swab	12/05/2018	imp	imp	1.51E+04
CPO078	*Klebsiella pneumoniae*	Wound swab	08/10/2012	kpc	kpc	5.83E+03
CPO079	*Citrobacter spp*	Rectal Swab	01/10/2017	kpc	kpc	9.73E+03
CPO080	*Klebsiella pneumoniae*	Urine	22/03/2014	kpc	kpc	1.22E+04
CPO081	*Klebsiella pneumoniae*	Rectal Swab	10/09/2017	kpc	kpc	3.48E+03
CPO082	*Citrobacter spp*	Rectal Swab	15/10/2017	kpc	kpc	3.45E+03
CPO083	*Klebsiella pneumoniae*	Urine	08/04/2015	kpc	kpc	7.61E+03
CPO084	*Serratia marcescens*	Rectal Swab	10/10/2017	kpc	kpc	8.66E+03
CPO085	*Klebsiella pneumoniae*	Rectal Swab	11/09/2017	kpc	kpc	3.98E+03

CPO086	*Klebsiella pneumoniae*	Rectal Swab	11/09/2017	kpc	kpc	1.82E+04
CPO087	*Escherichia coli*	Rectal Swab	01/08/2016	ndm	ndm	4.23E+03
CPO088	*Klebsiella pneumoniae*	Urine	25/12/2015	ndm	ndm	5.39E+03
CPO089	*Klebsiella pneumoniae*	Rectal Swab	18/12/2015	ndm	ndm	6.47E+03
CPO090	*Escherichia coli*	Bone (Tibia)	14/01/2015	ndm	ndm	3.69E+03
CPO091	*Klebsiella pneumoniae*	Throat swab	02/08/2016	ndm	ndm	3.66E+03
CPO092	*Klebsiella pneumoniae*	Rectal Swab	18/04/2015	ndm	ndm	1.19E+04
CPO093	*Klebsiella pneumoniae*	Urine	23/04/2015	ndm	ndm	4.26E+03
CPO094	*Klebsiella pneumoniae*	Rectal Swab	25/04/2015	ndm	ndm	2.22E+03
CPO095	*Proteus mirabilis*	Urine	07/02/2014	ndm	ndm	1.40E+03
CPO096	*Klebsiella pneumoniae*	Rectal Swab	04/12/2016	ndm	ndm	2.63E+03
CPO097	*Klebsiella pneumoniae*	Mouth Swab	29/01/2015	ndm	ndm	2.48E+03
CPO098	*Klebsiella pneumoniae*	Rectal Swab	20/04/2015	ndm	ndm	2.39E+03
CPO099	*Klebsiella pneumoniae*	Perinrum swab	10/05/2015	ndm	ndm	1.00E+04
CPO100	*Escherichia coli*	Vaginal swab	05/03/2015	ndm	ndm	1.40E+04
CPO101	*Escherichia coli*	Perinrum swab	09/04/2015	ndm	ndm	9.82E+03
CPO102	*Klebsiella pneumoniae*	Perinrum swab	09/12/2014	ndm	ndm	1.56E+04
CPO103	*Klebsiella pneumoniae*	Rectal Swab	20/04/2015	ndm	ndm	1.66E+04
CPO104	*Klebsiella pneumoniae*	Rectal Swab	15/04/2015	ndm	ndm	1.06E+04
CPO105	*Escherichia coli*	Faeces	23/11/2015	ndm	ndm	9.28E+03
CPO106	*Escherichia coli*	Rectal Swab	26/12/2015	ndm	ndm	7.51E+03
CPO107	*Klebsiella pneumoniae*	Perinrum swab	07/05/2015	ndm	ndm	6.29E+03

CPO108	*Klebsiella pneumoniae*	Rectal Swab	20/04/2015	ndm	ndm	1.85E+04
CPO109	*Klebsiella pneumoniae*	Sputum	23/03/2018	ndm	ndm	9.74E+03
CPO110	*Klebsiella pneumoniae*	Rectal Swab	29/04/2015	ndm	ndm	1.27E+04
CPO111	*Klebsiella pneumoniae*	Perinrum swab	07/05/2015	ndm	ndm	1.05E+04
CPO112	*Klebsiella pneumoniae*	Catheter Urine	02/07/2014	ndm	ndm	1.21E+04
CPO113	*Klebsiella pneumoniae*	Perinrum swab	09/12/2014	ndm	ndm	1.50E+04
CPO114	*Klebsiella pneumoniae*	Urine	08/03/2015	ndm	ndm	8.01E+03
CPO115	*Klebsiella pneumoniae*	Urine	10/04/2015	ndm	ndm	2.06E+04
CPO116	*Klebsiella pneumoniae*	Perinrum swab	19/04/2015	ndm	ndm	1.46E+04
CPO117	*Klebsiella pneumoniae*	Rectal Swab	18/04/2015	ndm	ndm	2.83E+04
CPO118	*Klebsiella pneumoniae*	Rectal Swab	17/05/2015	ndm	ndm	9.15E+03
CPO119	*Klebsiella pneumoniae*	Urine	23/04/2015	ndm	ndm	2.22E+04
CPO120	*Klebsiella pneumoniae*	Rectal Swab	25/04/2015	ndm	ndm	1.41E+04
CPO121	*Klebsiella pneumoniae*	Perineum swab	10/05/2015	ndm	ndm	8.60E+03
CPO122	*Klebsiella pneumoniae*	Rectal Swab	03/10/2015	ndm	ndm	2.08E+04
CPO123	*Klebsiella pneumoniae*	Rectal Swab	16/08/2015	ndm	ndm	1.96E+04
CPO124	*Klebsiella pneumoniae*	Wound swab	19/04/2015	ndm	ndm	6.44E+03
CPO125	*Klebsiella pneumoniae*	Rectal Swab	16/09/2015	ndm	ndm	1.68E+04
CPO126	*Klebsiella pneumoniae*	Rectal Swab	07/06/2015	ndm	ndm	1.64E+04
CPO127	*Klebsiella pneumoniae*	Rectal Swab	07/07/2015	ndm	ndm	2.29E+04
CPO128	*Klebsiella pneumoniae*	Rectal Swab	03/06/2015	ndm	ndm	6.54E+03
CPO129	*Klebsiella pneumoniae*	Rectal Swab	20/05/2015	ndm	ndm	1.18E+04

CPO130	*Klebsiella pneumoniae*	Rectal Swab	10/08/2015	ndm	ndm	7.64E+03
CPO131	*Klebsiella pneumoniae*	Perineum swab	12/07/2015	ndm	ndm	6.86E+03
CPO132	*Klebsiella pneumoniae*	Rectal Swab	21/07/2015	ndm	ndm	2.40E+04
CPO133	*Escherichia coli*	Rectal swab	29/06/2018	ndm	ndm	1.02E+04
CPO134	*Klebsiella pneumoniae*	Blood culture	23/09/2016	ndm	ndm	3.14E+04
CPO135	*Enterobacter cloacae*	Rectal swab	24/06/2018	ndm	ndm & kpc	5.75E+04
CPO136	*Escherichia coli*	Urine	12/03/2019	ndm	ndm	1.83E+04
CPO137	*Escherichia coli*	Rectal swab	28/01/2019	ndm	ndm	1.29E+04
CPO138	*Klebsiella pneumoniae*	Rectal swab	23/02/2019	ndm	ndm	2.41E+04
CPO139	*Klebsiella pneumoniae*	Rectal swab	25/03/2019	ndm	ndm	5.85E+04
CPO140	*Enterobacter cloacae*	Rectal swab	03/04/2019	ndm	ndm	1.24E+04
CPO141	*Enterobacter cloacae*	Rectal swab	21/01/2019	ndm	ndm	8.38E+03
CPO142	*Citrobacter freundii*	Abdomen	08/04/2019	ndm	ndm	1.34E+04
CPO143	*Klebsiella pneumoniae*	Urine	23/04/2015	ndm	ndm	1.11E+04
CPO144	*Klebsiella pneumoniae*	Right leg tissue	29/07/2015	ndm	ndm	7.75E+03
CPO145	*Klebsiella pneumoniae*	Abdomen	22/09/2016	ndm	ndm	1.55E+04
CPO146	*Escherichia coli*	Urine	04/05/2017	ndm	ndm	1.73E+04
CPO147	*Escherichia coli*	Catheter urine	06/06/2019	ndm	ndm	6.45E+03
CPO148	*Escherichia coli*	Rectal swab	12/01/2019	ndm	ndm	1.13E+05
CPO149	*Escherichia coli*	Rectal swab	27/05/2019	ndm	ndm	2.44E+05
CPO150	*Enterobacter cloacae*	Rectal swab	16/06/2016	ndm	ndm	2.72E+06
CPO151	*Klebsiella pneumoniae*	Rectal swab	16/05/2019	ndm	ndm	7.59E+04

CPO152	*Enterobacter cloacae*	Rectal swab	12/05/2018	ndm	ndm	2.48E+06
CPO153	*Enterobacter cloacae*	Rectal swab	22/04/2018	ndm	ndm	1.80E+06
CPO154	*Escherichia coli*	Rectal swab	18/08/2018	ndm	ndm	3.59E+05
CPO155	*Klebsiella pneumoniae*	Rectal swab	08/05/2019	ndm	ndm	1.87E+06
CPO156	*Enterobacter cloacae*	Rectal swab	12/05/2018	ndm	ndm	1.11E+05
CPO157	*Klebsiella pneumoniae*	Rectal swab	07/01/2019	ndm	ndm	1.54E+06
CPO158	*Klebsiella pneumoniae*	Rectal swab	12/01/2019	ndm	ndm	2.27E+06
CPO159	*Klebsiella pneumoniae*	Rectal swab	13/03/2019	ndm	ndm	3.15E+04
CPO160	*Klebsiella pneumoniae*	Rectal swab	27/05/2019	ndm	ndm	9.98E+04
CPO161	*Escherichia coli*	Rectal Swab	01/12/2014	ndm & oxa48	ndm & oxa48	1.70E+04
CPO162	*Escherichia coli*	Wound swab	14/03/2014	oxa48	oxa48	2.45E+04
CPO163	*Escherichia coli*	Rectal Swab	20/10/2017	oxa48	oxa48	3.76E+03
CPO164	*Citrobacter freundii*	Rectal Swab	06/01/2016	oxa48	oxa48	5.56E+03
CPO165	*Escherichia coli*	Urine	04/04/2015	oxa48	oxa48	2.48E+03
CPO166	*Klebsiella pneumoniae*	Rectal Swab	07/01/2016	oxa48	oxa48	1.50E+04
CPO167	*Escherichia coli*	Wound swab	25/11/2012	oxa48	oxa48	2.22E+03
CPO168	*Escherichia coli*	Blood culture	21/07/2013	oxa48	oxa48	1.17E+04
CPO169	*Serratia marcescens*	Bone (Tibia)	28/01/2015	oxa48	oxa48	6.25E+03
CPO170	*Citrobacter freundii*	Rectal Swab	13/12/2015	oxa48	oxa48	3.23E+03
CPO171	*Klebsiella pneumoniae*	Urine	02/07/2014	oxa48	oxa48	1.46E+03
CPO172	*Klebsiella pneumoniae*	Drain Fluid Abdomen	22/01/2015	oxa48	oxa48	4.55E+03
CPO173	*Escherichia coli*	Urine	04/04/2015	oxa48	oxa48	2.25E+04

CPO174	*Escherichia coli*	Rectal swab	28/06/2018	oxa48	oxa48	1.16E+04
CPO175	*Klebsiella pneumoniae*	Urine	08/05/2018	oxa48	oxa48	3.36E+04
CPO176	*Klebsiella pneumoniae*	Blood culture	13/06/2018	oxa48	oxa48	2.77E+04
CPO177	*Klebsiella pneumoniae*	Blood culture	15/10/2018	oxa48	oxa48	3.40E+04
CPO178	*Klebsiella pneumoniae*	Blood culture	21/02/2015	oxa48	oxa48	8.70E+03
CPO179	*Citrobacter freundii*	Rectal swab	14/01/2018	oxa48	oxa48	4.21E+04
CPO180	*Citrobacter freundii*	Urine	16/01/2018	oxa48	oxa48	3.12E+04
CPO181	*Klebsiella pneumoniae*	Rectal swab	04/03/2019	oxa48	oxa48	6.24E+04
CPO182	*Klebsiella pneumoniae*	Urine	30/08/2018	oxa48	oxa48	2.14E+04
CPO183	*Klebsiella pneumoniae*	Urine	23/04/2019	oxa48	oxa48	8.64E+04
CPO184	*Klebsiella pneumoniae*	Urine	01/06/2015	oxa48	oxa48	2.82E+05
CPO185	*Escherichia coli*	Wound swab	08/07/2015	oxa48	oxa48	1.58E+04
CPO186	*Klebsiella pneumoniae*	Urine	04/01/2016	oxa48	oxa48	9.71E+04
CPO187	*Citrobacter amalonaticus*	Rectal swab	18/01/2016	oxa48	oxa48	3.38E+04
CPO188	*Klebsiella pneumoniae*	Urine	08/02/2016	oxa48	oxa48	8.94E+05
CPO189	*Klebsiella pneumoniae*	Urine	08/02/2016	oxa48	oxa48	4.47E+05
CPO190	*Klebsiella pneumoniae*	Wound swab	01/07/2016	oxa48	oxa48	5.80E+05
CPO191	*Klebsiella pneumoniae*	Pleural fluid	10/07/2016	oxa48	oxa48	7.23E+05
CPO192	*Escherichia coli*	Rectal swab	15/08/2016	oxa48	oxa48	4.37E+04
CPO193	*Escherichia coli*	Urine	26/08/2016	oxa48	oxa48	2.14E+04
CPO194	*Klebsiella pneumoniae*	Urine	22/10/2016	oxa48	oxa48	1.35E+05
CPO195	*Escherichia coli*	Urine	27/11/2016	oxa48	oxa48	1.11E+04

CPO196	*Klebsiella pneumoniae*	Rectal swab	18/03/2017	oxa48	oxa48	5.28E+05
CPO197	*Escherichia coli*	Wound swab	18/04/2017	oxa48	oxa48	9.71E+03
CPO198	*Citrobacter freundii*	Urine	05/05/2017	oxa48	oxa48	5.71E+03
CPO199	*Klebsiella pneumoniae*	Wound swab	06/08/2017	oxa48	oxa48	1.29E+04
CPO200	*Klebsiella pneumoniae*	Abdomen	17/01/2018	oxa48	oxa48	1.73E+03
CPO201	*Klebsiella pneumoniae*	Rectal swab	22/01/2018	oxa48	oxa48	1.78E+03
CPO202	*Klebsiella pneumoniae*	Urine	27/01/2018	oxa48	oxa48	1.26E+05
CPO203	*Escherichia coli*	Rectal swab	22/04/2018	oxa48	oxa48	1.02E+06
CPO204	*Citrobacter freundii*	Rectal swab	18/08/2018	oxa48	oxa48	5.77E+05
CPO205	*Escherichia coli*	Rectal swab	07/01/2019	oxa48	oxa48	2.69E+05
CPO206	*Escherichia coli*	Rectal swab	12/01/2019	oxa48	oxa48	1.78E+04
CPO207	*Citrobacter freundii*	Rectal swab	11/02/2019	oxa48	oxa48	2.92E+06
CPO208	*Escherichia coli*	Rectal swab	13/03/2019	oxa48	oxa48	1.57E+04
CPO209	*Klebsiella pneumoniae*	Rectal swab	27/05/2019	oxa48	oxa48	2.45E+06
CPO210	*Klebsiella pneumoniae*	Rectal swab	08/05/2019	oxa48	oxa48	2.03E+06
CPO211	*Klebsiella pneumoniae*	Rectal swab	11/05/2019	oxa48	oxa48	2.41E+05
CPO212	*Klebsiella pneumoniae*	Rectal swab	16/06/2016	oxa48	oxa48	3.61E+05
CPO213	*Escherichia coli*	Rectal swab	16/05/2019	oxa48	oxa48	3.29E+04
CPO214	*Klebsiella pneumoniae*	Rectal swab	12/05/2018	oxa48	oxa48	1.21E+06
CPO215	*Klebsiella pneumoniae*	Rectal swab	22/04/2018	oxa48	oxa48	1.57E+06
CPO216	*Klebsiella pneumoniae*	Rectal swab	18/08/2018	oxa48	oxa48	4.18E+05
CPO217	*Klebsiella pneumoniae*	Rectal swab	07/01/2019	oxa48	oxa48	2.43E+06

CPO218	*Escherichia coli*	Rectal swab	12/01/2019	oxa48	oxa48	1.80E+05
CPO219	*Escherichia coli*	Rectal swab	11/02/2019	oxa48	oxa48	2.78E+03
CPO220	*Klebsiella pneumoniae*	Rectal swab	13/03/2019	oxa48	oxa48	3.75E+05
CPO221	*Escherichia coli*	Rectal swab	27/05/2019	oxa48	oxa48	5.40E+06
CPO222	*Klebsiella pneumoniae*	Rectal swab	08/05/2019	oxa48	oxa48	9.42E+03
CPO223	*Klebsiella pneumoniae*	Rectal swab	11/05/2019	oxa48	oxa48	3.71E+05
CPO224	*Citrobacter freundii*	Rectal swab	16/06/2016	oxa48	oxa48	7.02E+05
CPO225	*Escherichia coli*	Rectal swab	12/05/2018	oxa48	oxa48	8.26E+05
CPO226	*Klebsiella pneumoniae*	Rectal swab	22/04/2018	oxa48	oxa48	7.85E+05
CPO227	*Escherichia coli*	Rectal swab	18/08/2018	oxa48	oxa48	4.46E+05
CPO228	*Citrobacter freundii*	Rectal swab	07/01/2019	oxa48	oxa48	5.04E+05
CPO229	*Klebsiella pneumoniae*	Rectal swab	11/02/2019	oxa48	oxa48	2.57E+05
CPO230	*Klebsiella pneumoniae*	Rectal swab	13/03/2019	oxa48	oxa48	4.65E+05
CPO231	*Klebsiella pneumoniae*	Rectal swab	08/05/2019	oxa48	oxa48	4.16E+05
CPO232	*Klebsiella pneumoniae*	Rectal swab	11/05/2019	oxa48	oxa48	1.03E+05
CPO233	*Klebsiella pneumoniae*	Rectal swab	07/01/2019	oxa48	oxa48	2.01E+06
CPO234	*Klebsiella pneumoniae*	Rectal swab	12/01/2019	oxa48	oxa48	6.13E+04
CPO235	*Escherichia coli*	Rectal swab	11/02/2019	oxa48	oxa48	2.54E+05
CPO236	*Klebsiella pneumoniae*	Rectal swab	13/03/2019	oxa48	oxa48	2.26E+05
CPO237	*Escherichia coli*	Rectal swab	27/05/2019	oxa48	oxa48	2.22E+05
CPO238	*Escherichia coli*	Rectal swab	11/05/2019	oxa48	oxa48	8.02E+05
CPO239	*Klebsiella pneumoniae*	Rectal swab	16/06/2016	oxa48	oxa48	2.90E+05

CPO240	*Klebsiella pneumoniae*	Rectal swab	16/05/2019	oxa48	oxa48	2.93E+05
CPO241	*Escherichia coli*	Rectal swab	22/04/2018	oxa48	oxa48	5.33E+05
CPO242	*Enterobacter cloacae*	Rectal swab	18/08/2018	oxa48	oxa48	8.86E+04
CPO243	*Klebsiella pneumoniae*	Rectal swab	16/06/2016	oxa48	oxa48	4.46E+06
CPO244	*Klebsiella pneumoniae*	Rectal swab	18/08/2018	oxa48	oxa48	2.11E+06
CPO245	*Enterobacter spp*	Rectal swab	27/05/2019	oxa48	oxa48	4.32E+04
CPO246	*Pseudomonas aeruginosa*	Wound swab	25/03/2015	vim	vim	2.28E+03
CPO247	*Citrobacter freundii*	Rectal Swab	02/04/2016	vim	vim	1.33E+04
CPO248	*Enterobacter cloacae*	Bone (Tibia)	14/01/2015	vim	vim	5.66E+03
CPO249	*Pseudomonas aeruginosa*	Sputum	01/11/2013	vim	vim	5.19E+03
CPO250	*Enterobacter cloacae*	Bone (Tibia)	14/01/2015	vim	vim	2.95E+04
CPO251	*Klebsiella pneumoniae*	Rectal swab	11/02/2019	vim	vim	1.49E+05
CPO252	*Klebsiella pneumoniae*	Rectal swab	08/05/2019	vim	vim	1.90E+05
CPO253	*Klebsiella pneumoniae*	Rectal swab	11/05/2019	vim	vim	1.39E+05
